# Gait Rehabilitation in Ambulant Diplegic Children Using Botulinum A Injection and Ankle Weights

**DOI:** 10.1155/2022/6544813

**Published:** 2022-12-30

**Authors:** Nahla M. Ibrahim, Hatem Galal Abdallah Ibrahim, Tarek Alsayad, Mahrous I. Seddeek, Talal A. Dawa, Adel Ibrahim Azzam, Abd El-Hamid Gaber, Ashraf Abdelkader

**Affiliations:** ^1^Department of Physical Therapy for Paediatrics, Faculty of Physical Therapy, Cairo University, Giza, Egypt; ^2^Department of Rheumatology and Rehabilitation, Faculty of Medicine (Boys), Al-Azhar University, Cairo, Egypt; ^3^Department of Pediatrics, Faculty of Medicine (Boys), Al-Azhar University, Cairo, Egypt; ^4^Department of Neurology, Al-Azhar University, Faculty of Medicine (Boys), Cairo, Egypt; ^5^Department of Clinical Pharmacology, Faculty of Medicine, Menoufia University, Menoufia, Shebin Al Kawm, Egypt

## Abstract

**Objectives:**

Standing and walking serve an individual's basic needs to move from place to place, and both are the most common activities that people do daily. So, this study aims to investigate the combined effect of botulinum A injection and ankle weight on excessive knee flexion in diplegic children with crouch gait.

**Methods:**

Sixty children with spastic diplegia walking with a crouch gait were included in this study. They were divided equally into three groups (twenty in each): group *A* received classical gait rehabilitation, group *B* received the same gait training while adding ankle weights, and group *C* received the same as group *A* and *B* plus botulinum A injection. The modified Ashworth scale (MAS) and Hoffman reflex/Myogenic response (*H*/*M* ratio) were used to evaluate the spasticity of the hamstring and gastrocnemius muscles, while two-dimension gait analysis was used to record knee flexion angles during gait. The assessment was held one day before starting the treatment and after completing three months of the treatment program.

**Results:**

There was no significant difference between groups before treatment regarding all measured variables. group *A* revealed a statistically nonsignificant improvement after treatment. Patients in group *B* showed significant improvement after treatment for both knees regarding the *H*/*M* ratio and MAS, which was reflected in the right and left knee range of motion at initial contact (*P* values 0.030 and 0.001, respectively) and midstance (*P* values 0.030 and 0.006, respectively). However, more significant improvement was detected regarding all studied variables in both knees after treatment in group C patients with a *P* value <0.001.

**Conclusion:**

The combination of botulinum A injection and ankle weights was more effective in controlling excessive knee flexion in diplegic children with a crouch gait.

## 1. Introduction

Cerebral palsy (CP) is characterized by primary neuromuscular deficits such as spasticity and muscle weakness, as well as secondary musculoskeletal problems such as bony malformations and contractures [1]. Although roughly 70% of those children can walk, they all have different degrees of deviations associated with this function [2]. Insults that occur in the brain prevent the normal development of postural control mechanisms, resulting in many impairments [3]. Diplegia is the most common type of CP. It accounts for approximately 32% of that population in which the lower extremities are more affected than the upper extremities. The most common physical impairments in diplegic children are gait deviations such as crouching and scissoring, which are the most common constricting gait patterns in children with diplegia [4]. Crouching is a gait pattern that many people with spastic diplegia have that is characterized by excessive flexion of the hips, knees, and ankles during the stance phase of gait. Walking in this manner is inefficient and can result in joint pain [5] and bone deformities [1]. One of the possible causes of this gait is hypertonicity of the calf, hamstrings, or hip flexors. Other factors that have been identified include poor balance, abnormalities of the feet, reduced proprioception, and muscle weakness [6].

Gait training is an effective treatment exercise that leads to improvement in walking patterns with a low risk of injury to the musculoskeletal system due to its nonharmful effect on the human body [7]. However, it requires a long time to gain satisfactory results because walking as a traditional exercise has the disadvantage of being boring [8]. Different ways to increase exercise benefits during the same amount of time have been introduced to save time and enhance the efficiency of exercise [9]. Examples include walking while holding a dumbbell in each hand or wearing a sandbag around each ankle [10].

Wearing ankle weights throughout the day can improve the calf, quad, and hip muscles since the patient has to work harder to flex and move the leg. To our knowledge, the precise benefits of wearing ankle weights during daily activities have not been studied. In particular, Barnett et al. reported that ankle weights are useful for ankle stability and should be minimized in order to maximize walking velocity [11]. Furthermore, Odéen and Knutsson observed that employing ankle weights while walking helped lower muscular tone in patients with spastic paraplegia [12].

Spastic patients who were injected with botulinum toxin A have dose-dependent partial chemo-denervation, resulting in reduced muscle activity lasting roughly 12–16 weeks. Increased flexibility and muscle stretch are possible due to decreased tone and inhibition of spastic reflexes, which are thought to promote muscle elongation [13]. In one clinical trial of children with spastic equines, injection into the gastrocnemius led to muscle lengthening for a period of 24 weeks after the injection [14]. Several reports showed the gait parameters changed through the significant improvement of ankle dorsiflexion at initial contact after injection into calf muscles [15]. However, to the best of our knowledge, the effect of botulinum toxin type A injections into the hamstring on the muscle-tendon length and gait kinematics in children walking with flexion knees has rarely been studied [16–18].

Spasticity is associated with abnormalities in muscle tone and overactive stretch reflexes in children with CP [19]. Both muscular weakness and spasticity are considered to be significantly detrimental to function in children with CP [20]. With strong supportive evidence for the use of strength training to target muscular weakness and, similarly, the use of botulinum toxin type A for spasticity management and improved function, should we be applying strength training concurrently with botulinum toxin type A treatment to further improve gait function for children with diplegia? With the combination of both botulinum toxin type A and strength training in the form of ankle weight, those children can have improvements in muscle control with a reduction in spasticity. Furthermore, the primary purpose of this study was to evaluate the combined effect of spastic muscle injection with botulinum toxin type A and gait training physiotherapy applying ankle weight on gait in children with diplegic CP.

## 2. Materials and Methods

### 2.1. Study Design

A randomized controlled trial was conducted from January to April 2021 in accordance with the Code of Ethics of the World Medical Association (Declaration of Helsinki). Before the commencement of evaluation or treatment procedures, written consent was obtained from the children's parents for both participation and publication of the study results. This study was conducted after obtaining approval from the Research Ethical Committee Center, Faculty of Cairo University, with the number P.T. REC/012/003212. The study was registered at clinicaltrials.gov with the following identifier: NCT05294874 URL: https://register.clinicaltrials.gov.

### 2.2. Randomization

Sixty-nine participants met the enrollment criteria of the study. Enrolled children were block-randomized into 3 equal groups (*A*, *B*, and *C*) by an independent person (who was not involved in the research team) using a computer Microsoft Excel program (computer-generated random numbers in each group according to a predetermined ratio of 1 : 1 : 1). At the time of randomization, parents were blinded to the grouping, and the statistical analyst was blinded to the treatment given to each group. Participants in group *A* received classical gait training, participants in group *B* received classical gait training while adding ankle weight, and participants in group *C* received gait training with ankle weight after injection. Unfortunately, nine participants (3 in each group) declined to finish the whole treatment session (as they could not attend the sessions regularly) before collecting the results after treatment. Therefore, sixty children (twenty in each group) were statistically analyzed at the end of the study.

**Figure 1:**
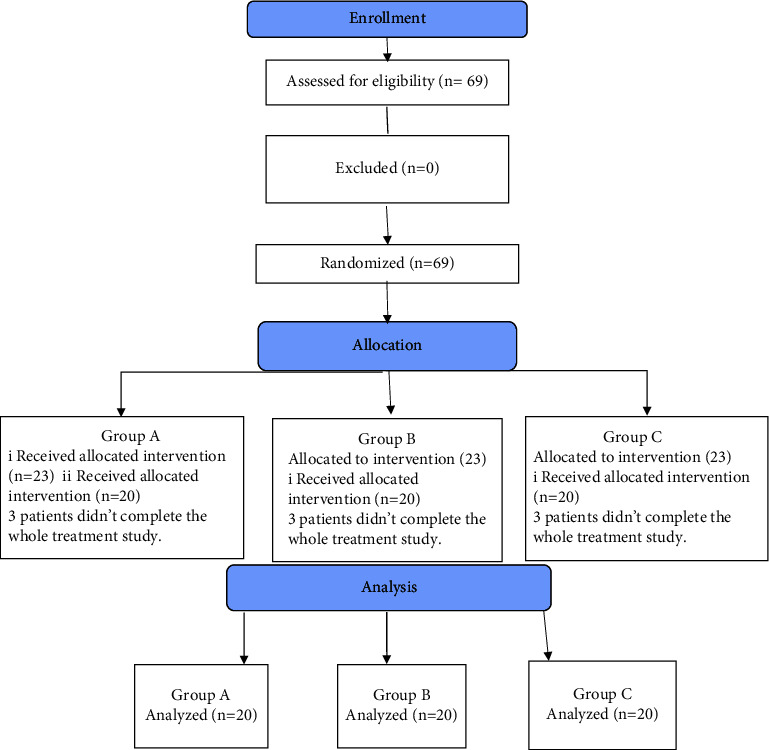
Participant' flow chart.

Participants' flow chart (Figure 1).

### 2.3. Sample Size

It was calculated using the *G*^*∗*^power software (version 3.1.9.4) computer program to reduce a type II error and increase the power based on a one-way analysis of variance (ANOVA) test with the comparison of the 3 studied groups. A sample size of 66 children was calculated using a significance level of 0.05, an effect size of 0.40, a power (1 − *β*) of 0.80, and an a priori set at 0.05. Considering that we were losing some participants during the study, before collecting post-treatment results, we anticipated a dropout rate and aimed to finally include 69 patients (23 in each group).

### 2.4. Subjects

Sixty-nine children with spastic diplegia had crouch gait, their ages ranged from five to ten years old. They could understand simple verbal instructions and had a spasticity grade of 1+ or 2 in both calf and hamstring muscles according to the modified Ashworth scale [21] (MAS), a level I or II on the Gross Motor Function Classification System (GMFCS) [22]. Criteria for exclusion included fixed contractures or deformities in the lower limbs, surgical orthopedic intervention or botulinum A injection, or the use of neuromuscular blockers in the previous 6 months before starting the study.

A physical therapy exercise program was conducted 3 sessions/week for successive 3 months at the Faculty of Physical Therapy Pediatric Outpatient Clinic, Cairo University.

### 2.5. Outcome Measures

#### 2.5.1. Evaluation of Spasticity

- Modified Ashworth scale
- It was used for the evaluation of muscle tone in the hamstring and gastrocnemius muscles of both lower limbs before and after 3 months of treatment. The test was carried out by one examiner to avoid variability in the recorded measures.
- For hamstring muscles, children were asked to lie in a prone position, keeping their hips in a neutral position with their ankles at the edge of a plinth and relaxing; then the knee was moved into extension through the full range of motion passively 3 times to evaluate the degree of muscle resistance. The score on the scale was detected according to the degree of resistance to passive movement while extending the knee and the ankle.
- The gastrocnemius was evaluated supine, with the hips in the neutral position and the ankle relaxed in plantar flexion; the ankle was then moved passively (while the patient was relaxed) with the maximum possible dorsiflexion for a maximum of three times, and the degree of resistance to the movement was rated on the MAS.
- The scale scores from 0 to 4, in which 0 refers to no increase in muscle tone, while 4 was recorded when the limb was rigid in flexion or extension [23].
- *H*/*M* ratio
- Recording of *H* and *M* responses from the right and left gastrocnemius muscles was performed using a surface electromyography (EMG) system (Medelec, Sapphire 4ME, Surrey, United Kingdom). Children were asked to relax in a prone position, keeping their feet out of the plinth. Before the assessment, the skin was cleaned with a piece of cotton before electrode placement to avoid skin resistance. The first electrode (an active surface electrode) was placed over the highest circumference point of the gastrocnemius muscle, and the second electrode (an indifferent electrode) was taped over the proximal part of the Achilles tendon. Submaximal stimulation was used to elicit an *H* response while supramaximal stimulation of the posterior tibial nerve was used to induce an *M* (motor) response in the gastrocnemius muscle using the same stimulating and recording electrodes [24].

### 2.6. Evaluation of Knee Range of Motion during Gait

#### 2.6.1. Two-Dimensional Gait Analysis

It was used to evaluate knee ROM during initial contact and midstance subphases of the stance phase of gait for both lower limbs for children in the three groups before and after treatment.

For the evaluation of gait, children were wearing comfortable light clothing and walking in a straight line at their usual comfortable speed. The laboratory environment for data collection was previously organized into a two-dimensional reference system. Specific markers were placed on the target anatomical points of the lower limbs (greater trochanter of the femur, center of the knee joint, and lateral aspect of the ankle joint). The line coordinates of markers in the greater trochanter of the femur and the knee joint center are defined as the thigh vector. The leg vector was defined by the coordinates of the center knee joint up to the lateral ankle joint.

To record the gait movements, a single camera (Panasonic NV-GS180®) with an acquisition frequency of 30 Hz (frame/s) was placed perpendicularly to the reference system. After recording, the images were analyzed by The Tracker Video Analysis and Modeling Tool, built on the open-source physics Java framework. It is designed to model and analyze the motion of objects in video by overlaying simple dynamical models directly onto the videos and extracting the angles.

The gait cycle starts with successive contacts of the heel of the right foot with the floor. The flexion-extension ROM of both knees during initial contact and midstance gait subphases was recorded as the angle between the thigh and leg vector before and after treatment for all groups. An average of three records was collected for analysis.

#### 2.6.2. Treatment Procedures

Each physiotherapy session lasted an hour; it was performed three times a week on alternate days for three months.

Physical therapy exercise program for children in the following three groups:

(1) Children in all groups underwent a physiotherapy program in the form of flexibility exercises for the hip flexors, knee flexors, and ankle plantar flexors of both lower limbs. Balance exercises during standing and walking. Progressive resisted exercise for hip extensors, knee extensors, and ankle dorsi flexors.
(2) Gait training exercises
  - Walking forward, backward, and sideways
  - Walking with a stepper
  - Walking across different obstacles (different sizes of rolls, wedges, and blocks).

#### 2.6.3. Ankle Weights

Children in group *B* had received gait training exercises as in group *A* in addition to adding ankle weight at both sides. Ankle weights that corresponded to 2% to 3% of the individual's body weight (sandbags) were attached at 5 cm above the left and right ankle joints using Velcro-type straps [25].

#### 2.6.4. Botulinum A Injection

Following baseline evaluation, group *C* patients were injected and started a gait training program using ankle weight on the third day following injection. Injection of hamstring and gastrocnemius muscles at both sides of botulinum toxin type A (Botox; Allergan, Inc., Irvine, CA) with 100 units of *Clostridium botulinum* A per vial, human serum albumin, and sodium chloride in a sterile, vacuum-dried form without preservatives were given to group *C* patients. The dosing and injection procedures were carried out in accordance with (Russman et al., 1997) guidelines. To achieve a concentration of 5 U/0.1 mL, each vial was reconstituted with 2 mL of sterile saline. The maximum dose for any single injection was not more than 200 U. Based on the dosage calculation of 3–6 U/kg, a syringe was used to draw an aliquot. Prior to giving the shots, no anesthesia was employed. Using 23–26-gauge needles and an insulin syringe, injections were given under sterile circumstances. A needle was introduced through the fascia into the proximal one-third of the muscle after the target muscle was palpated while being stretched passively (approximate region of the motor end plates). Each side's gastrocnemius and hamstring muscles (Semimembranosus, Semitendinosus, and Biceps femoris) were injected [26].

### 2.7. Statistical Analysis

The SPSSS computer package version 25.0 (IBM SPSS, Armonk, NY: IBM Corp., USA) was used for statistical analysis of the data. For descriptive statistics, the mean ± SD was used for quantitative variables that were normally distributed, while the number and percentage were used for qualitative variables. In analytic statistics, the Chi-square test was used to assess differences in the frequency of qualitative variables, while the one-way ANOVA test was applied to assess differences in means of quantitative variables between the three groups, with Bonferroni post hoc correction to determine where the significance specifically exists, and paired samples *A t*-test was used to assess differences in the means of quantitative variables within the same group before and after therapy. The statistical methods were verified, assuming a significant level of *p* < 0.05 and a highly significant level of *p* < 0.001.

## 3. Results

The study included 60 children with spasticity fulfilling the inclusion and exclusion criteria, divided into 3 equal groups with no significant differences in age (*p*=0.605), gender (*p*=0.626), and body mass index (BMI) (*p*=0.124). Before therapy, no significant differences were detected between groups regarding MAS (*p*=0.077 Rt & *p*=0.086 Lt), H/M ratio (*p*=0.585 Rt & *p*=0.069 Lt), ROM at initial contact right and left (0.067, 0.128), and ROM at midstance Rt and Lt (0.083, 0.070) in both the right and left knees. After therapy, they showed a significant decrease (improvement) in both groups *B* and *C* compared to group A in both the right and left knees. However, the improvement was significantly more obvious in group *C* compared to group *A* (for all parameters, *p* < 0.001). Similarly, within groups *B* and *C*, they showed significant reduction (improvement) after therapy (*H*/*M* ratio Rt: *p*=0.005 for *B* and *p* < 0.001 for *C*), whereas within group *A*, they showed insignificant improvement after therapy (for all parameters, *p* > 0.05) (Table 1).

Before therapy, no significant differences were detected between the studied groups regarding MAS in both the right (*P*=0.077) and left (*P*=0.086) knees, with more children having a score of “2” and “1+.” After therapy, a significant increase (improvement) was observed in children with a score of “0” in the right knee (10%, 25%, and 65%; *P* < 0.001) and in the left knee (10%, 35%, and 70%; *P* < 0.001) in groups *A*, *B*, and *C*, respectively (Table 2).

Within group *A*, no significant improvement (increased scores “0” and “1”) was observed in MAS after therapy in both right (*P*=0.259) and left (*P*=0.236) knees. Within group *B*, a significant improvement (increased score “0” & decreased scores “1+” and “2”) was observed in MAS after therapy; (score “0” increased from 15% to 25%, scores “1+” and “2” decreased from 45% to 20% to 0%, respectively, *P* < 0.001) in the right knee and (score “0” increased from 10% to 35% and score “1+” decreased from 45% to 0%, *P*=0.002) in the left knee. Similar significant improvement was observed within group *C* after therapy; (score “0” increased from 0% to 65%, and scores “1+” and “2” decreased from 40% to 15% to 0%, respectively, *P* < 0.001) in the right knee and (score “0” increased from 5% to 70%, score “1+” and “2” decreased from 45% to 10% to 0%, respectively, *P* < 0.001) in the left knee (Table 3).

## 4. Discussion

In our study, we investigated the effect of adding ankle weight in the gait training program on the degree of knee flexion of children with crouch gait after botulinum A injection of the hamstring and gastrocnemius muscles. MAS and *H*/*M* ratio were used to evaluate the degree of spasticity, while two-dimension gait analysis was used to investigate their effect on knee flexion angle during the initial contact and midstance phases of gait. The assessment was done before and after 3 successive months of the treatment program.

Results revealed nonsignificant differences between groups regarding all measured variables before treatment. Group *A* showed little improvement in gait pattern but with no statistically significant difference concerning spasticity and knee flexion angle. In group *B*, adding ankle weights to the lower limbs during gait training showed significant improvement in decreasing spasticity and knee flexion angles during gait. The most significant improvement in controlling spasticity was more obvious in group *C*, which was reflected in decreasing excessive knee flexion during initial contact and midstance subphases of gait in children with crouch gait. So, it can be concluded that using ankle weight during gait after botulinum injection of the spastic hamstring and gastrocnemius muscles was effective in controlling the degree of knee flexion in diplegic children.

A normal gait is a form of human locomotion that is characterized by comfortable and economical smooth repetitive movements of the joints [27]. While walking, different degrees of flexion and extension movements occur at the knee. At initial contact, in which feet touch the floor, at the start of the stance phase, the knee joint can show a maximum flexion of 18°. At the swing phase, with lower limb transposition (from 40 to 70% of the gait cycle), the knee flexes up to 70° [28].

Our results that concluded a significant decrease in the *H*/*M* ratio as a reflection of the decreased muscle spasticity after injection in diplegic children in group *C* are also in agreement with those of (Suputtitada), who reported significantly decreased spasticity in 10 spastic diplegic and hemiplegic CP children after botulinum A injection [29].

Injection of the hamstring and gastrocnemius by botulinum type A resulted in a decreased degree of spasticity at the knee, which was approved by decreasing the values of *H*/*M* as well as the score level of MAS ratio after treatment. These results are comparable to those reported by (Koman et al.) in that participants who were treated with botulinum A had greater improvement in the mean score for gait pattern and ankle position at initial contact [30].

In normal children, the degree of knee flexion or extension is almost near or at zero at the start of the stance phase by initial foot contact. In contrast, children with cerebral palsy showed excessive knee flexion at the point of starting a heel strike due to spasticity or shortening of the knee flexors in both lower limbs [31]. This comes to agree with Kim et al. [32, 33], who found that cerebral palsy children have excessive knee flexion and limited knee extension more than normal children during the stance phase.

Different studies considered the effect of weight on hemiparetic children. To our knowledge, there was no study that evaluated the effect of loading on diplegic children. In a study of hemiparetic children by Simão et al., in which they compared the angular variables before and immediately after gait training, they noticed that 60% of the lower limb weight promoted a more pronounced increase in the angular variables of hip and knee flexion, thus resulting in a multijoint motor strategy in response to the resistance imposed during the swing phase [34]. This strategy has also been observed in healthy individuals in response to external perturbations by increasing the height of the trajectory of the foot during the swing phase. Moreover, a strong correlation was observed between the amount of load added and the activation of hip flexor muscles [35].

Now, strength training is well recognized as an effective intervention method for improving muscular strength in children with CP [36]. There is an increase in the number of studies that confirm that following strength training in those children, significant muscle strength improvements were observed and reflected gains in motor functions such as walking [36–38] and balance [39]. McNee and colleagues reported that in children with CP, improvements in strength are associated with an increase in volume, muscle size, and strength after 5 to 10 weeks of strength training [40]. Whilst strength training targets muscular weakness, botulinum A is a proven treatment option for spasticity control for children with CP [41]. In this study, the outcomes of spasticity reduction and controlling knee ROM and their effect on gait functions were overwhelmingly positive.

### 4.1. Study Limitations

Assessment of muscle strength was a limitation of our study, as was the short follow-up period after 3 months of treatment to evaluate the posteffect of treatment. Also, the small number of participants enrolled in this study, even if statistically significant differences were obtained between groups, might be seen as a shortcoming. So, results should be interpreted with caution. In addition, we hypothesized an effect size of 0.40 without a prior pilot study that may affect the magnitude of the difference between the sets of data. So, the small sample size was a limitation of this study. So, we encourage further research to explore this with a bigger sample of participants.

## 5. Conclusion

Injections of botulinum A into spastic muscles in addition to adding ankle weights to the gait training program may provide an effective nonsurgical technique for decreasing excessive knee flexion and so improving the quality of gait in children with crouch gait.

## Figures and Tables

**Table 1:** Comparison of H/M ratio, ROM at initial contact and at midstance pre- and post-therapy in both right and left knees between the studied groups and within each group.

Variables		Group *A*	Group *B*	Group *C*	*P*-value
		(*n* = 20%)	(*n* = 20%)	(*n* = 20%)	
Age		6.59 ± 1.58	6.17 ± 1.39	6.23 ± 1.19	0.605

Gender	Male	9 (45)	11 (55)	12 (60)	0.626
	Female	11 (55)	9 (45)	8 (40)	

BMI		17.5 ± 0.46	17.68 ± 0.57	17.32 ± 0.58	0.124

*H*/*M* ratio right	Pre	0.37 ± 0.06	0.36 ± 0.07	0.35 ± 0.06	0.585
	Post	0.34 ± 0.06	0.30 ± 0.07	0.25 ± 0.04	<0.001^*∗*^^1,2,3^

*P* value		0.082	0.005^*∗*^	<0.001^*∗*^	

H/M ratio left	Pre	0.37 ± 0.07	0.33 ± 0.03	0.34 ± 0.04	0.069
	Post	0.34 ± 0.07	0.29 ± 0.06	0.25 ± 0.03	<0.001^*∗*^^1,2,3^

*P* value		0.099	0.041^*∗*^	<0.001^*∗*^	

ROM initial contact right	Pre	46.7 ± 9.6	41.6 ± 8.6	40.6 ± 8.0	0.067
	Post	44.5 ± 7.4	36.4 ± 8.5	28.6 ± 9.6	<0.001^*∗*^^1,2,3^

*P* value		0.071	0.030^*∗*^	<0.001^*∗*^	

ROM initial contact left	Pre	45.6 ± 11.7	41.6 ± 8.7	39.8 ± 6.2	0.128
	Post	42.4 ± 10.0	35.2 ± 8.0	29.0 ± 7.0	<0.001^*∗*^^1,2^

*P* value		0.105	0.001^*∗*^	<0.001^*∗*^	

ROM midstance right	Pre	33.6 ± 14.4	26.6 ± 11.7	25.8 ± 9.5	0.083
	Post	31.0 ± 13.6	21.1 ± 11.4	13.3 ± 6.4	<0.001^*∗*^^1,2^

*P* value		0.086	0.030^*∗*^	<0.001^*∗*^	

ROM midstance left	Pre	34.1 ± 15.5	26.3 ± 11.2	26.1 ± 8.8	0.070
	Post	32.3 ± 14.9	21.0 ± 11.6	14.3 ± 7.3	<0.001^*∗*^^1,2^

*P* value		0.115	0.006^*∗*^	<0.001^*∗*^	

^1^: significance between group *A* and group *B*. ^2^: significance between group *A* and group *C*. ^3^: Significance between group *B* and group *C*. ^*∗*^: significant. *P* value, 0.001 BMI: body mass index, ROM: range of motion, *H*/*M*: Hoffman/myogenic.

**Table 2:** Comparison between groups regarding MAS of both right and left knees pre- and post-therapy.

Variables		Group *A*	Group *B*	Group *C*	Total	*P*-value
		*n* = 20 (%)	*n* = 20 (%)	*n* = 20 (%)	*n* = 60 (%)	
MAS preright	0	0 (0)	3 (15)	0 (0)	3 (5)	0.077
	1	6 (30)	4 (20)	9 (45)	19 (31.7)	
	1+	6 (30)	9 (45)	8 (40)	23 (38.3)	
	2	8 (40)	4 (20)	3 (15)	15 (25)	

MAS preleft	0	2 (10)	2 (10)	1 (5)	5 (8.3)	0.086
	1	6 (30)	9 (45)	8 (40)	23 (38.3)	
	1+	5 (25)	9 (45)	9 (45)	23 (38.3)	
	2	7 (35)	0 (0)	2 (10)	9 (15)	

MAS postright	0	2 (10)	5 (25)	13 (65)	20 (33.3)	<0.001^*∗*^
	1	5 (25)	15 (75)	7 (35)	27 (45)	
	1+	9 (45)	0 (0)	0 (0)	9 (15)	
	2	4 (20)	0 (0)	0 (0)	4 (6.7)	

MAS postleft	0	2 (10)	7 (35)	14 (70)	23 (38.3)	<0.001^*∗*^
	1	4 (20)	13 (65)	6 (30)	23 (38.3)	
	1+	11 (55)	0 (0)	0 (0)	11 (18.3)	
	2	3 (15)	0 (0)	0 (0)	3 (5)	

^*∗*^: significant. *P* value <0.001, MAS: modified Ashworth scale.

**Table 3:** Comparison of MAS pre- and post-therapy within each group in both Rt and Lt knees.

Variables		Group *A*		Group *B*		Group *C*	
		*n* = 20 (%)		*n* = 20 (%)		*n* = 20 (%)	
		Pre	Post	Pre	Post	Pre	Post
MAS right	0	0 (0)	2 (10)	3 (15)	5 (25)	0 (0)	13 (65)
	1	6 (30)	5 (25)	4 (20)	15 (75)	9 (45)	7 (35)
	1+	6 (30)	9 (45)	9 (45)	0 (0)	8 (40)	0 (0)
	2	8 (40)	4 (20)	4 (20)	0 (0)	3 (15)	0 (0)

*P* value		0.259		<0.001^*∗*^		<0.001^*∗*^	

MAS left	0	2 (10)	2 (10)	2 (10)	7 (35)	1 (5)	14 (70)
	1	6 (30)	4 (20)	9 (45)	13 (65)	8 (40)	6 (30)
	1+	5 (25)	11 (55)	9 (45)	0 (0)	9 (45)	0 (0)
	2	7 (35)	3 (15)	0 (0)	0 (0)	2 (10)	0 (0)

*P* value		0.236		0.002^*∗*^		<0.001^*∗*^	

^*∗*^: significant. *P* value <0.001, MAS: modified Ashworth scale.

## Data Availability

Data that support the findings of this study are available upon request from the corresponding author. They are not publicly available due to privacy and ethical restrictions.
